# Overcoming Physiological Bottlenecks of Leaf Vitality and Root Development in Cuttings: A Systemic Perspective

**DOI:** 10.3389/fpls.2020.00907

**Published:** 2020-06-30

**Authors:** Uwe Druege

**Affiliations:** Erfurt Research Centre for Horticultural Crops (FGK), University of Applied Sciences Erfurt, Erfurt, Germany

**Keywords:** internal quality, senescence, adventitious rooting, plant development, phytohormones, primary metabolism, environment, modeling

## Abstract

Each year, billions of ornamental young plants are produced worldwide from cuttings that are harvested from stock plants and planted to form adventitious roots. Depending on the plant genotype, the maturation of the cutting, and the particular environment, which is complex and often involves intermediate storage of cuttings under dark conditions and shipping between different climate regions, induced senescence or abscission of leaves and insufficient root development can impair the success of propagation and the quality of generated young plants. Recent findings on the molecular and physiological control of leaf vitality and adventitious root formation are integrated into a systemic perspective on improved physiologically-based control of cutting propagation. The homeostasis and signal transduction of the wound responsive plant hormones ethylene and jasmonic acid, of auxin, cytokinins and strigolactones, and the carbon-nitrogen source-sink balance in cuttings are considered as important processes that are both, highly responsive to environmental inputs and decisive for the development of cuttings. Important modules and bottlenecks of cutting function are identified. Critical environmental inputs at stock plant and cutting level are highlighted and physiological outputs that can be used as quality attributes to monitor the functional capacity of cuttings and as response parameters to optimize the cutting environment are discussed. Facing the great genetic diversity of ornamental crops, a physiologically targeted approach is proposed to define bottleneck-specific plant groups. Components from the field of machine learning may help to mathematically describe the complex environmental response of specific plant species.

## Introduction

Plant propagation is the initial process of producing ornamental crops that already sets the first benchmark for the whole cultivation process by determining the quality of the young plant. Many ornamental plant species are propagated vegetatively by rooting of shoot tip cuttings. Depending on the plant genotype, the developmental stage of the cutting, and specific constellation of environmental factors, impaired vitality of cuttings or insufficient adventitious root (AR) formation in the stem base can cause leaf losses and dying of cuttings or failures in rooting and delayed or uneven root formation among individuals that impairs synchronous subsequent growth. Furthermore, the increasing demand on sustainability of young plant production requires improved propagation protocols that should provide maximum utilization of the genetically determined endogenous potential of the crop.

Propagation of ornamental plants by cuttings can be characterized as follows:

(1) It involves control of plant development in highly equipped ecosystems, that are mainly determined by technically controlled environmental inputs, for example in greenhouses, growth and storage rooms etc. I propose the term “technoecosystems” for such systems.
(2) Environmental inputs are highly dynamic, because propagation involves a complex chain of subsequent processes that occur in different environments. Stock plant cultivation and harvest of cuttings occurs in diverse climatic regions far remote from the market of young plants and is followed by packaging, storage, transport, sticking and cultivation of cuttings close to the market.
(3) The chain is continuously changing, because new priorities in the society and developing technologies provide new demands and options. Examples are the increasing demand for saving energy and reducing pollution, the changing lighting technology and the up-coming rooting systems that are compatible with transport logistics and sticking robots.
(4) There is a great genetic diversity of plants that are propagated by cuttings. This results from the high number of species used in floriculture and also from the high breeding activities bringing more than thousand new cultivars to the European market year by year. For example, in 2018, 140 applications for protection of *Chrysanthemum* varieties were submitted to the Community Plant Variety Office (Anonymus, 2019).

Considering this complex and highly dynamic situation, there is the need for a general concept, which helps to understand the limitation of and how to optimize cutting propagation in a specific system of interest, as characterized by the particular plant genotype and the environmental inputs that can be technically controlled during the specific chain. In this context, endogenous quality attributes of a cutting should reflect its current capacity to develop into a young plant. Recently, comprehensive review articles have provided detailed views on the molecular, hormonal and metabolic control of AR formation (Steffens and Rasmussen, 2016; Druege et al., 2019; Lakehal and Bellini, 2019). In this perspective article, the essential knowledge concerning this process is brought into context with the problem of leaf senescence and abscission, and further integrated into a systemic approach that faces the demands and the complex environment of ornamental horticulture. A physiologically based model of crucial processes determining the success in cutting propagation, their linkages to critical environmental inputs as well as those physiological outputs that can be used to monitor cutting function are introduced.

## Systemic Model of Propagation by Cuttings

### The Cutting Function

**FIGURE 1:**
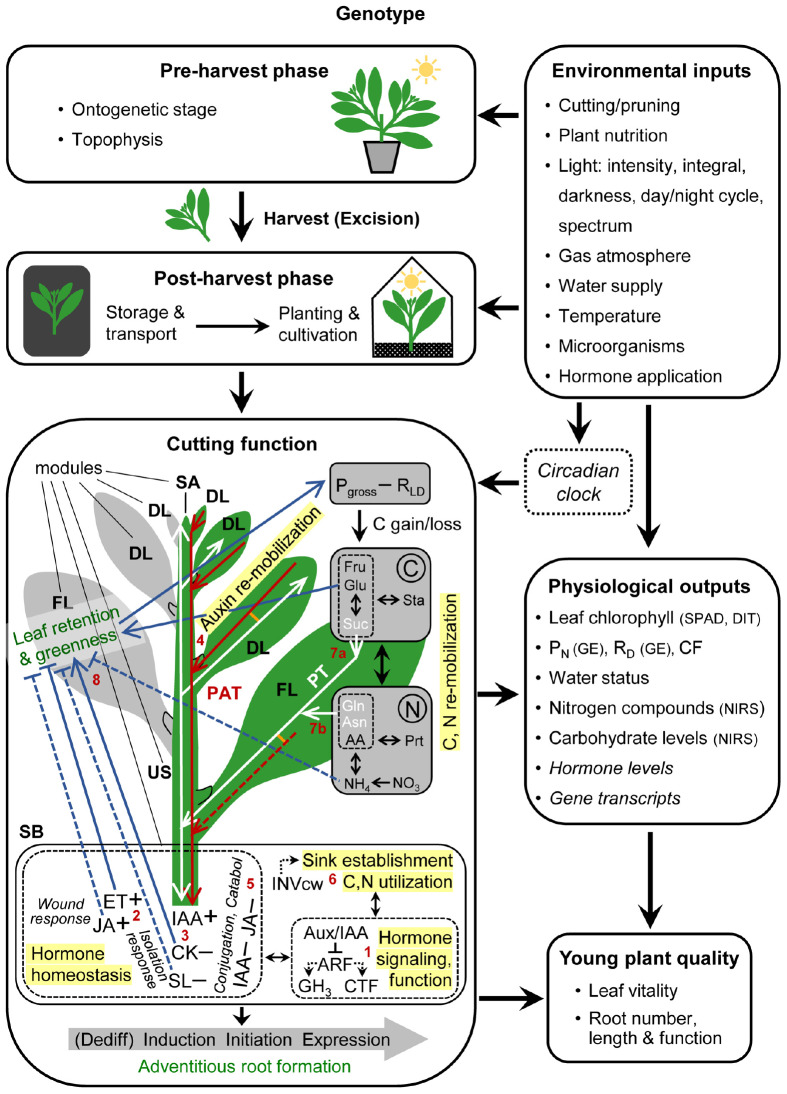
Systemic, process-based model of clonal propagation by utilization of adventitious root formation in cuttings. Red and white lines and arrow heads indicate the pathways and directions of polar auxin transport (PAT) and phloem transport (PT), respectively. Lines in orange indicate PAT-PT connections. Blue lines ending with arrow heads or crossbars indicate positive or negative effects of increasing concentrations and of leaf retention and greenness, respectively. Plus versus minus signs indicate increased versus decrease of hormone concentrations, respectively, in response to excision. Red numbers indicate the bottlenecks explained in the text. *Italic letters* mark those physiological outputs, which are currently not accessible to routine analysis under practical conditions. Abbreviations between brackets indicate currently available measuring principles. AA, amino acids; ARF, auxin responsive factors; Aux/IAA, auxin/indole-3-acetic acid repressors; Asn, asparagine; CTF, cell fate regulating transcription factors; Catabol, catabolism; CK, cytokinins; Dediff, dedifferentiation; CF, chlorophyll fluorescence; DIT, digital image technology; DL, developing leaf; ET, ethylene; FL, fully developed leaf; GE, gas exchange analysis; GH3, Gretchen Hagen 3; Gln, glutamine; Glu, glucose; IAA, indole-3-acetic acid; INWcw, cell wall invertase; JA, jasmonic acid; NIRS, near infrared reflectance spectroscopy; P_N_, net photosynthesis; P_gross_, gross photosynthesis; Prt, proteins; R_D_, dark respiration; R_LD_, light and dark respiration; SA, shoot apex; SB, stem base; SL, strigolactones; SPAD, Soil Plant Analysis Development-chlorophyll meter; Sta, starch; suc, sucrose. Further explanations see Box 1 and the text.

The cutting is considered as functional unit in the center of the propagation system (Figure 1). Leaf retention and greenness and AR formation are the two targets of cutting function that determine the final quality of the young plant. Depending on the initial status of the AR source cells [cells, in which adventitious rooting starts (Druege et al., 2019)], their dedifferentiation may be involved first to gain competence for AR induction. Induction of AR competent cells leads to cell specification toward subsequent initiation (ending with the formation of primordia) and final expression of ARs from the stem base (SB). There, homeostasis of wound-responsive ethylene (ET) and jasmonic acid (JA), of the auxin indole-3-acetic acid (IAA), cytokinins (CK) and strigolactones (SL), and hormone signaling and function are core processes regulating AR formation, which is further dependent on sink establishment and C and N utilization. Fully developed leaves (FL) constitute important source tissues for N and C re-mobilization, while the developing leaves (DL) and shoot apex (SA) compete with the stem base (SB) for these resources. IAA accumulation in the SB is dependent on auxin re-mobilization from the upper shoot with polar auxin transport (PAT) as main driving process. The changed hormone homeostasis of the cutting may affect leaf senescence and abscission. Further explanation is given in Box 1.

**Box 1. Targets, modules and processes of cutting function**

**Leaf retention and greenness** and **adventitious root formation** in the stem base are the **two targets** of cutting function that determine the final **quality of the young plant**, while **physiological outputs** can be used to monitor the cutting performance.

**Plant genotype and environmental inputs**, which act pre-harvest during the stock plant phase and after the harvest of cuttings, control the function of cuttings. This may also involve the circadian clock. The function of the cutting is further subject to the **ontogenetic stage** of the stock plant and to the cutting position within the stock plant (**topophysis**), which determines the specific developmental stage as well as the source-sink and hormonal status of the cutting tissues at time of harvest.

**Important functional modules** are the **stem base (SB)** as zone of root regeneration and of C and N utilization, the **upper stem (US)** as transport unit for all compounds and as intermediate storage unit of C and N, the **fully developed leaves (FL)** as source organs for providing C, N and possibly auxin, and the **developing leaves (DL)** and **shoot apex (SA)** as competitive utilization sinks for C and N and as potential source (DL) or sink (SA) of auxin.

In the SB, **hormone homeostasis**, **signaling and function** control the **dedifferentiation (Dediff)**, **induction**, **initiation** and **expression** of adventitious roots, with Aux/IAA proteins, auxin responsive factor (ARF) transcription factors, GH3 proteins, and cell fate regulating transcription factors (CTF) such as GRAS, AP2/ERF and WOX as important components. **Sink establishment and C and N utilization** provide building blocks, energy and metabolic signals. Wound-induced accumulation of ethylene (ET) and jasmonic acid (JA), a decrease in levels of root-sourced cytokinins (CK) and strigolactones (SL) due to isolation from the stock plant, and accumulation of IAA contribute to dedifferentiation and induction of ARs. IAA accumulation in the stem base is dependent on **auxin re-mobilization** from the upper shoot, with DL and FL as potential auxin sources and **polar auxin transport (PAT)** as important process of translocation.

Depending on the sensitivity of the leaves, systemic effects of the changed plant hormone homeostasis may control **leaf retention and greenness** by affecting **leaf senescence** and **abscission**, with ethylene (ET) and cytokinins (CK) as classical trigger and inhibitors of leaf senescence or abscission, respectively. Whether the principally known agonistic effects of jasmonic acid (JA) and strigolactones (SL) on leaf senescence have relevance to cuttings, requires further investigation. High sugar levels, particularly of glucose can counteract leaf senescence/abscission, possibly via depressed ET signalling.

The earliness of **establishment and the strength of the new utilization sink in the stem base** as compared to the competing sink in the upper shoot limits the influx of C and N resources into the stem base. Activity of cell wall invertase (INVcw) as crucial driver of initial sink activity may be under positive control by IAA and JA.

**C and N re-mobilization** from the upper shoot, particularly from fully developed leaves is an important process for the delivery of sugars and amino acids (AA) to the utilization sink in the stem base, with sucrose (Suc), asparagine (Asn) and glutamine (Gin) as important components of **phloem transport (PT)**.

The balance **of gross photosynthesis (P_gross_)** and **respiration** in the light and in the dark (R_LD_) determines the **carbon gain or loss** of the leaf and thus its **carbohydrate pool**, with starch (Sta) and Suc as intermediate storage and main export fraction, respectively, while glucose (Glu) and Fructose (Fru) are locally utilized.

Light-dependent **assimilation of nitrate and ammonium**, that have been initially delivered from the root system of the stock plant, determines one influx of N into the **AA and protein (Prt) pools**, while competing with the sugar pool for C skeletons. Under prolonged dark conditions of whole cuttings that deplete the carbohydrate pool of leaves, leaf metabolism is readjusted towards a survival strategy, resulting in **accumulation of AA**, while proteolysis contributes to this process. If the released AAs are further catabolized to free **ammonium**, this may trigger leaf senescence.

### The Bottlenecks

Depending on the plant genotype and configuration of environmental inputs at stock plant and cutting level, different processes may constitute the bottleneck (B) of cutting function (Figure 1).

#### B1: General Auxin Responsiveness in the Stem Base

If auxin signaling is generally low in the candidate AR source cells and not stimulated by the changed hormone homeostasis after cutting (see B2), no or few ARs will be formed. Cuttings, particularly of woody ornamentals such as *Rosa* or *Hydrangea macrophylla*, can exhibit low rooting capacity in dependence on the specific genotype (Dubois and Vries, 1991) or when they have been collected from mature parts of the stock plant (Galopin et al., 1996). The underlying principles are only fragmentary understood. A recent genome-wide association study of 95 rose genotypes indicated functions of the auxin and ET signal transduction (see also B2) in the diversity of AR formation, pointing to one auxin response factor (ARF), to cell fate regulating transcription factors (CTF), e.g., of the WOX- and GRAS-families that act down-stream of auxin, and to one positive regulator of the ET response pathway (Nguyen et al., 2020). In some forest tree species, the maturation-related decline in rooting capacity was related to divergences in expression of specific ARFs or CTF of the GRAS-family (Diaz-Sala, 2014, 2019). In *Eucalyptus grandis*, the maturation-induced decline in auxin-induced rooting was related to disturbed microtubule remodeling, which functional relevance was proven by chemical manipulation (Abu-Abied et al., 2014). However, whether these relationships are based on bottleneck functions of the auxin signal transduction *per se* or rather reflect upstream limitations of cellular plasticity that is under further control of microRNAs and epigenetic factors (Poethig, 2013; Diaz-Sala, 2014; Druege et al., 2019) requires further investigation (see also B2).

#### B2: Initial ET-JA-CK Interaction in the Stem Base

If AR source cells require dedifferentiation before AR induction, early wound induced accumulation of ET and JA, together with CKs may contribute to this process by enhancing auxin responsiveness (Druege et al., 2019) (see B1).

#### B3: Early Rise of Auxin/CK-SL Ratio in the Stem Base

If AR source cells are root competent (see also B2), AR induction is dependent on local accumulation of IAA and further supported by a decrease in levels of the antagonistic CKs and SLs. Cutting off from (a) the root-ward auxin-drain and (b) the root-sourced delivery of CKs and SLs initiates these changes which may be supported by local IAA biosynthesis or release from auxin conjugates (Druege et al., 2019).

#### B4: Auxin Re-mobilization From the Upper Shoot

In intact plants, IAA is synthesized in young expanding leaves and is in the stem either transported root-ward by polar auxin transport (PAT) in xylem parenchyma and cambium cells or co-transported in the phloem associated with assimilate transport (AT) (Kramer and Bennett, 2006; Petrasek and Friml, 2009; Leyser, 2011; van Rongen et al., 2019), while in leaves both pathways can be interconnected (Cambridge and Morris, 1996; Figure 1). These auxin routes are supplemented with a low conductance and less polar “connective auxin transport,” linking the PAT route to the surrounding tissues (Bennett et al., 2016; van Rongen et al., 2019). In shoot tip cuttings, PAT has important functions in auxin translocation toward the SB and, depending on the plant genotype and environmental condition, cutting leaves of different age may constitute important auxin sources (Guerrero et al., 1999; Garrido et al., 2002; Ahkami et al., 2013; Yang et al., 2019; Figure 1). Wounding and isolation of cuttings may trigger specific auxin biosynthetic pathways in addition to the pathways of the intact plant (Chen et al., 2016), possible via action of JA (Zhang et al., 2019).

#### B5: Late Decrease in Auxin and JA Levels in the Stem Base

Initiation and expression of ARs requires a decrease of IAA and probably also of the physiologically active conjugate of JA after the induction phase. Obviously, upregulation of specific GH3 proteins that function as acyl acid amido synthetases conjugating IAA or JA to specific AAs has an important role for this dynamic (Druege et al., 2019; Lakehal and Bellini, 2019). IAA level can be reduced also by catabolism, with oxidation as important route that can be triggered by JA (Lakehal et al., 2019; Figure 1).

#### B6: Sink Establishment and C and N Utilization in the Stem Base

Temporal courses of carbohydrates and amino acids (AA) in the stem base of cuttings during rooting (Ahkami et al., 2009) and the positive response of AR formation to applications of sugars and AA reveal that AR formation utilizes both resources (Orlikowska, 1992; Takahashi et al., 2003; Correa et al., 2005; Schwambach et al., 2005; Yasodha et al., 2008). After excision of cuttings, the transport route of these resources has to be directed toward the developing ARs, while the DLs and SA constitute competitive sink organs. Provided C or N sources are not the limiting factors (see B7), the strength of the new utilization sink in the SB limits the influx of C and N into the SB (Figure 1 and Box 1). This is determined by the activities of invertases, in particular cell wall invertase which can be up-regulated by IAA and JA (Roitsch and Gonzalez, 2004; Ahkami et al., 2009, 2013; Albacete et al., 2011; Agulló-Antón et al., 2014).

#### B7: Surplus of C and N in Source Leaves

If the sink in the SB is sufficiently high to attract resources (B6), their influx can be limited by the source capacity of the upper shoot, with the fully developed leaves (FL) as main source organs (Figure 1). Depending on the plant genotype and the particular environmental conditions during the chain, the magnitudes of either the C (*B7a*) or N source (*B7b*) may limit rooting (Druege et al., 2000, 2004; Zerche et al., 2016). The available surplus of carbohydrates in cutting leaves depends on the initial carbohydrate level at time of planting and the current C gain during cutting cultivation (Rapaka et al., 2005), while current photosynthesis and C gain of cuttings is obviously dependent on plant species (Druege and Kadner, 2008; Klopotek et al., 2012). The level in leaves of total sugars, particularly of sucrose, during the rooting period reflects the carbohydrate source limitation of rooting in cuttings (Rapaka et al., 2005; Druege and Kadner, 2008; Klopotek et al., 2010).

#### B8: Hormone Sensitivity of Leaves

Senescence or abscission of leaves can impair the visual quality and vitality of cuttings and may finally cause decay and loss of whole cuttings. Changed concentrations in cuttings particularly of ET, JA, and CK in response to their excision have consequences for leaf senescence and/or abscission when the changed hormone homeostasis meets the respective responsiveness of the leaf (Figure 1 and Box 1). Both, the change in hormone levels and the leaf response are dependent on the plant genotype. Stimulation of ET biosynthesis by wounding or other stresses may promote leaf senescence in cuttings of sensitive plant species, particularly when packaging and storage of cuttings entraps the ET in the surrounding air (Müller et al., 1998; Rapaka et al., 2007a, b; Leatherwood et al., 2016). Depletion of CK in cuttings in response to the cut off from the root system can contribute to post-harvest leaf senescence, since CK application rescued leaf greenness antagonizing ET effects (Mutui et al., 2005). Observed agonistic effects of JA and SL on leaf senescence in intact plants (Ueda and Kusaba, 2015; Zhuo et al., 2020) may also be relevant to cuttings. Free ammonium can promote leaf senescence (Britto and Kronzucker, 2002), whereas high sugar levels obviously protect cutting leaves from senescence or abscission, while agonistic and antagonistic effects on ET signaling may be involved, respectively (Yanagisawa et al., 2003; Druege et al., 2004; Rapaka et al., 2007a, b; Druege and Kadner, 2008; Li et al., 2019).

### Critical Environmental Inputs

Diverse environmental factors at stock plant and cutting level modify the function of cuttings and thereby control the quality of the young plant as final output of the system (Figure 1). Mechanisms of plant nutritional factors and effects of controlled atmosphere storage are largely unexplored. In addition to the effects of light intensity and integral and of CO_2_ on the C gain of cuttings, distinct effects of light spectrum may involve changes of auxin homeostasis and signaling (Ruedell et al., 2015; Christiaens et al., 2019). Dark-induced carbohydrate depletion in cuttings can impair AR formation via reduced C source. However, if high photosynthetic activity of cuttings after the dark period allows fast recovery of the C source, dark storage can promote AR formation (Klopotek et al., 2010). In addition to the enhancement of AA levels in cuttings following carbohydrate depletion (Box 1), dark storage of cuttings may promote sink establishment in the stem base via up-regulation of invertases (Klopotek et al., 2016) and stimulate auxin signaling by enhanced auxin mobilization from the upper shoot (Yang et al., 2019). There is indication from growth analyses of petunia plants, that also the circadian clock, which coordinates plant metabolism with the environment, has important functions in carbon allocation toward roots (Feller et al., 2015). The mechanisms underlying the recently observed stimulation of AR formation by targeting of water to the rooting zone (sub-misting) when compared to overhead misting are not understood. There is indication that beneficial effects of inoculations of specific root endophytes, such as arbuscular mycorrhizal fungi or *Serendipita indica* (former *Piriformospora indica*) at stock plant or cutting level on leaf vitality and AR formation may involve changed C source and hormone signaling in cuttings. However, effects of such inoculations are highly variable and even include negative effects on AR formation, when comparing different environmental conditions, modes of inoculation and plant genotype (Druege et al., 2006, 2007; Justice et al., 2018). This highlights the need for future research on the underlying mechanisms and key factors in the plant-microorganism-abiotic environment continuum. More details about the modes of action of the distinct environmental inputs (Figure 1) on cutting function consider also findings and concepts of Kadner and Druege (2004), Husen and Pal (2007), Rapaka et al. (2008), Bredmose and Nielsen (2009), Agulló-Antón et al. (2011), Osterc and Stampar (2011), Franken (2012), da Costa et al. (2013), Bauerfeind et al. (2015), Rasmussen et al. (2015), Otiende et al. (2017), Zhang et al. (2017), Ferrari et al. (2018), Peterson et al. (2018), Taylor and Hoover (2018), Cho et al. (2019), Heide (2019), Sanchez et al. (2020) and are summarized in Supplementary Table S1.

### Physiological Outputs

Distinct physiological parameters of cuttings can be used to monitor their function, which means to determine the efficiency of the processes contributing to leaf vitality and AR formation in the stem base (Figure 1). These parameters may be used to define the functional capacity of the cuttings as important internal quality attribute of cuttings or to adjust the environmental factors toward optimum cutting performance.

Even though the distinct effects of different levels and locations of water supply (top vs. bottom) on rooting of cuttings are unclear (Supplementary Table S1), monitoring the water status of cuttings can help to avoid water deficit induced limitation of leaf function (e.g., the reduction of carbon gain by stomatal closure) and AR formation at early rooting stages and to acclimate cuttings to moderate stress conditions at later rooting stages when ARs have already been formed. Water status of cuttings can be assessed by measuring transpiration *viz*. the water loss through the leaves, stomatal conductance representing the guard cell vapor conductivity, and the relative water content or water potential both reflecting the dehydration state of leaves or whole cuttings (Aminah et al., 1997; LeBude et al., 2005; Wilkerson et al., 2005). In addition to direct measurements of such parameters e.g., by use of gas exchange cuvettes, porometers etc., indirect methods and tools for real-time sensing of plant water status are increasingly available. These use for example stem thickness, leaf compressibility and thermal or spectral signatures of leaves (Millan-Almaraz et al., 2010; Steppe, 2015; Gerhards et al., 2019; Gautam and Pagay, 2020). However, the use of thermal signatures, which utilizes the dependency of leaf temperature on transpiration is complex in top-misting systems, where leaf temperature is decreased with each mist application.

Leaf chlorophyll content of leaves can be used to monitor leaf senescence and may also limit the C gain of cuttings by restriction of P_gross_. One of the most frequently used non-invasive tools for chlorophyll analysis in plant leaves is the Soil Plant Analysis Development (SPAD) (Figure 1) chlorophyll meter (SPAD-502, Konica Minolta, Osaka, Japan), that measures leaf transmittance in the red (650 nm; the measuring wavelength) and infrared (940 nm; a reference wavelength), while the output gives a relative value that is proportional to the chlorophyll content of the leaf (Ling et al., 2011). This tool has also been used in studies on ornamental crops, where nitrogen content as building block of chlorophyll was mostly the target (Bracke et al., 2019 and references therein) and also in some descriptive studies on cuttings (Rufai et al., 2016; Chater et al., 2017). Very high correlations can be found between chemically analyzed chlorophyll concentrations and SPAD values within a certain plant species as shown for Arabidopsis (Ling et al., 2011). However, the plant matrix obviously can affect these relationships as reflected by lower correlations, when ten different species of leafy vegetables were compared (Limantara et al., 2015). Digital image technology (DIT, Figure 1) is one alternative to analyze leaf color and also selectively chlorophyll contents, for example by use of the Red Green Blue color model. Such technologies may even provide a better estimation of chlorophyll than SPAD (Riccardi et al., 2014) and are further compatible to smartphones (Rigon et al., 2016).

Infrared gas analyzers can be used to determine the current carbon gain of cuttings or cutting leaves under the condition of measurement, which may, however, differ from the real conditions of rooting depending on the used measuring system (Druege and Kadner, 2008; Klopotek et al., 2012; Currey and Lopez, 2015; Tombesi et al., 2015). Use of chlorophyll fluorescence technology may provide information about the functional integrity of the photosynthetic apparatus. Quantum yield of PSII or quenching parameters in cutting leaves have already been used to determine their photosynthetic function as dependent on light acclimation (Rapaka et al., 2005), plant genotype (Druege and Kadner, 2008) and duration and temperature of dark storage (Faust and Enfield, 2010). Considering their relevance to the carbon gain and their stress response, online measurements of both leaf gas exchange and photosystem operating efficiency may be combined to optimize dynamic greenhouse control regimes during rooting of cuttings.

Considering that sugars and of AA in source tissues of cuttings reveal important fractions for the crucial C and N re-mobilization, analysis of their levels provides important information about the C and N source limitation of rooting (Druege et al., 2004; Rapaka et al., 2005; Zerche and Druege, 2009; Tombesi et al., 2015; Zerche et al., 2016). The N source availability in cuttings as affected by nitrogen fertilization of stock plants and by dark incubation is reflected not only by the levels of AA but also by the soluble organic nitrogen fraction (sum of amide- and amino-N) (Zerche et al., 2019). Lohr et al. (2016, 2017) have recently established protocols for non-invasive quantification of (a) soluble nitrogen fractions and total nitrogen in cuttings and (b) total non-structural carbohydrates, starch and total sugars (sum of glucose+fructose+sucrose) in cutting leaves by use of near infrared reflectance spectroscopy (NIRS). These methods provide a new basis for the control of cutting quality as affected by their C and N source availability during the propagation chain. Considering the progress in NIRS measuring systems during recent years (Ishikawa et al., 2014) and the potential of hyperspectral imaging for detection of individual metabolites in plant tissues (Vergara-Diaz et al., 2020), more selective non-invasive monitoring of the cutting metabolome should become possible in future.

Since hormone levels in the cutting tissues have decisive functions in leaf senescence and AR formation, their analysis can discover important bottlenecks of cutting function. Whereas commercial systems are available to measure ET emission by plants in closed environments such as storage packages or boxes (Caprioli and Quercia, 2014; Tolentino et al., 2018), analysis of plant hormones in plant tissues requires cutting edge laboratory equipment (Novak et al., 2017) and highly skilled analysts. Polymerase chain reaction (PCR) is standard in detection of plant pathogens (Lau and Botella, 2017) and Reverse Transcriptase (RT) PCR is a frequently used tool in human medicine to detect the expression of critical genes, e.g., in cancer diagnosis (Sager et al., 2015). To my knowledge, up to date no RNA-based tools are available for routine determination of internal quality of plant products such as cuttings. Nevertheless, considering that environmentally and genetically mediated AR formation in cuttings can depend on plant hormones, especially of IAA or the transcript levels of signaling components such as *ARF* and *GH3* genes (Ruedell et al., 2015; Yang et al., 2019), RNA-based quantitative diagnostic tools seem to have a high potential for next-generation functional diagnostics in cutting propagation. Considering the great diversity of ornamental plant species, such an approach must either be species-specific or focus on conserved DNA, respective RNA sequences that have same functions in several plant species.

## Outlook

The high complexity of highly dynamic environmental inputs in young plant production by cuttings and responding metabolic and hormonal pathways (Figure 1) is a challenge for the development of general rules or even mechanistic models to support a process-based management of cutting propagation. However, in a targeted approach the relevance of each potential bottleneck can be analyzed for selected plant species or cultivars and those can be grouped according to their identified bottlenecks. Further, the recently discussed concept of “plant perceptron” that implements components from the field of machine learning (Scheres and van der Putten, 2017) may help to mathematically describe these processes for model plant species. In such an approach, the early responding plant hormones JA, ET or IAA and the Aux/IAA-ARF modules (Figure 1) would constitute important environment-responsive factors of the so-called “top-hidden” and “bottom-hidden” layers of the information-processing system, respectively, that are weighted and integrated in the “output layer,” where the transcription of genes that control cell division, expansion and fate is regulated (Scheres and van der Putten, 2017). Because the cutting function provides the core of the conception (Figure 1), the presented model is open for and may stimulate the use of new environmental inputs to control propagation by cuttings. Some of the discussed physiological outputs are currently utilizable only for researchers and experimental conditions, because their monitoring requires high technical input and very specific skills. However, we can expect that the continuous technological progress, e.g., in the application of molecular tools will open more possibilities in future for the propagation industry to utilize physiological and molecular outputs of cuttings for optimizing the young plant production chain. Nevertheless, there will be the challenge to compromise between the specificity of targets, which increases with zooming from the crop to individual cuttings to individual organs such as FL or SB, and the relevance to the whole crop.

## Data Availability Statement

All datasets generated for this study are included in the article/Supplementary Material, further inquiries can be directed to the corresponding author.

## Author Contributions

UD wrote the article.

## Conflict of Interest

The author declares that the research was conducted in the absence of any commercial or financial relationships that could be construed as a potential conflict of interest.

## Supplementary Material

TABLE S1 Modes of action of environmental inputs on cutting function.
