# Supplementary material for: Overcoming Physiological Bottlenecks of Leaf Vitality and Root Development in Cuttings: A Systemic Perspective
Source: Front Plant Sci. 2020 Jun 30;11:907. doi: 10.3389/fpls.2020.00907 (PMC7340085; doi:10.3389/fpls.2020.00907)
Supplement: TABLE S1 — Modes of action of environmental inputs on cutting function. [file Data_Sheet_1.pdf]

Supplementary Material

Druege - leaf and root development cuttings

Table S1: Modes of action of environmental inputs on cutting function

Environmental input	Mode of action	References
Cutting and pruning	The frequency of harvesting, the position of cuttings within the stock plant and pruning of stock plants modify the homeostasis and signaling of plant hormones and the source/sink balance of the cuttings.	Husen and Pal, 2007; Osterc and Stampar, 2011; Diaz-Sala, 2014; Tombesi et al., 2015; Otiende et al., 2017; Heide, 2019
Plant nutrition	Nutrition of stock plants and of cuttings has complex but largely unexplored influences on rooting. Enhanced N supply to stock plants may stimulate adventitious root (AR) formation in cuttings via the enhanced N source promoting N re-mobilization and via stimulated auxin signaling in the stem base, but it lowers the carbohydrate pool (C source), starting with starch.	Druege et al., 2000, 2004, 2019; Zerche et al., 2016; Yang et al., 2019
Light intensity and integral	Acclimation of stock plants to high light enhances maximum photosynthesis but decreases the photosynthesis of cuttings under low light. Non-saturated increase in light intensity and daily light integral at stock plant and cutting level enhances the C gain of cuttings. Higher net photosynthesis, carbohydrate levels and availability of sucrose in source leaves provide a higher source of C, which can be translocated to the stem base, there favoring formation and growth of ARs.	Druege et al., 2004; Rapaka et al., 2005; Klopotek et al., 2012; Zerche and Druege, 2009; Currey and Lopez, 2015; Tombesi et al., 2015
Darkness	Dark storage of cuttings causes depletion of the C source in the cuttings. The reduced carbohydrate levels can impair AR formation and stimulate leaf senescence. Dark storage can stimulate AR formation, when sufficient photosynthesis after planting allows fast recovery of the carbohydrate levels. Obviously, dark-stimulated re-mobilization of amino acids from source leaves, enhanced sink competitiveness of the stem base against the upper shoot via upregulation of cell wall invertase and stimulated auxin accumulation and signaling are involved in positive dark effects on rooting.	Druege et al., 2004; Rapaka et al., 2005; Klopotek et al., 2011, 2012, 2016; Zerche et al., 2016; Yang et al., 2019
Day/night cycle	The day/night cycle affects the developmental phase of the stock plant in dependence on its genetically determined photoperiodic response type. Enhanced competence for flower induction in cuttings reduces the competence for AR induction while changed hormone levels and decreased auxin responsiveness may be involved.	Rasmussen et al., 2015
Light spectrum	Enhancing far red light at stock plant and red light at cutting level can promote AR formation in cuttings, while blue light perception has additional influences and enhanced PAT, auxin accumulation and signaling may be involved.	Ruedell et al., 2015; Cho et al., 2019; Christiaens et al., 2019

Gas atmosphere	Enhancing CO_2 in the air above ambient level can strongly enhance net photosynthesis and carbon gain of cuttings under light and thus may promote AR formation, if the C source is the limiting factor. Despite the well-known and frequently utilized positive effects of lowered O_2 and increased CO_2 levels in the atmosphere during dark storage on quality of fruits and vegetables, effects of such treatments on cuttings are largely unexplored. Increasing the CO_2 concentrations plus decreasing the O_2 concentrations in the controlled atmosphere (CA) of dark stored cuttings of the CAM plant *Kalanchoe blossfeldiana* strongly enhanced stomatal conductance of cutting leaves. After planting of the CA-stored cuttings, AR formation was similar to immediately planted fresh cuttings, but the specific response of rooting to the CA conditions was not investigated.	Klopotek et al., 2012; Bredmose and Nielson, 2009
Water supply	Water supply to cuttings is critical for maintaining their water balance which is challenged by the lost root system. Overhead misting is frequently applied, which frequency and duration modifies vapor pressure deficit of the air and leaf temperature, both affecting stomatal conductance and transpiration of leaves that can be critical to rooting. Recent studies revealed clear benefits of sub-mist targeted to the developing roots to AR development and growth, even when the vapor pressure deficit was much greater than in the overhead misting systems and net photosynthesis was not affected. Side effects of enhanced root zone temperature in the sub-mist systems may enhance resource (C and N) utilization in the rooting zone. Considering the water deficit response of ABA and its inhibitory role in cell division, reduced ABA levels in the misted stem bases or other hormonal events may contribute to such positive effects.	Le Bude et al., 2005; Wilkerson et al., 2005; da Costa et al., 2013; Peterson et al., 2018; Sanchez et al., 2020
Temperature	Non-saturated rise in rooting zone temperature favors AR formation in cuttings which can be explained by the fact that sink strength and metabolic processes of C utilization in roots are positively related to root zone temperature. The effect of air temperature on net photosynthesis of cuttings is dependent on the light level and the plant genotype. When gross photosynthesis of the cuttings is low, selective lowering of air temperature during rooting may enhance net photosynthesis, the carbon balance and rooting, provided root zone temperature is sufficiently high. This can be explained by a dominant influence of respiration, that decreases with lowering temperature and possibly also by a reduced competing sink in the cold-exposed upper shoot.	Wilkerson et al., 2005; Druege and Kadner, 2008; Klopotek et al., 2012; Bauerfeind et al., 2015; Ferrari et al., 2018
Microorganisms	Application of specific root endophytes such as arbuscular mycorrhizal fungi or *Serendipita indica* (former *Piriformospora indica*) to stock plants or to cuttings at time of planting can enhance the leaf vitality and intensity of AR formation. There is indication, that increased carbohydrate source availability or changed plant hormone homoeostasis and signaling in the host plants or cuttings may contribute to such responses.	Druege et al., 2006; Druege and Franken, 2007; Franken, 2012; Justice et al., 2018

Hormone application	Application of auxins can enhance AR formation. Treatments of cutting leaves and the stem base can be effective, while high concentrations are inhibitory and pulse treatments can restrict the auxin peak to the induction phase. Associated earlier cell division and establishment of new cell clusters and higher activities of invertases in the stem base as well as advanced peaks of soluble proteins and valleys of sugars and starch in the phloem of auxin-treated cuttings reveal that AR induction as well as sink establishment and C- and N-utilization in the stem base is stimulated by external auxin supply. Blocking of ethylene perception e.g. by application of methylcyclopropene during dark storage of cuttings can stimulate ethylene biosynthesis, which probably reflects the interruption of the negative feedback loop of ethylene biosynthesis in vegetative tissues (autoinhibiton).	Kadner and Druege, 2004; Rapaka et al., 2007b, 2008; Agullo-Anton et al., 2011, 2014; Rasmussen et al., 2015; Leatherwood et al., 2016; Zhang et al., 2017; Taylor and Hoover, 2018
